# Digital pathology and artificial intelligence in translational medicine and clinical practice

**DOI:** 10.1038/s41379-021-00919-2

**Published:** 2021-10-05

**Authors:** Vipul Baxi, Robin Edwards, Michael Montalto, Saurabh Saha

**Affiliations:** 1 Bristol Myers Squibb, Princeton, NJ USA; 2 PathAI, Boston, MA USA

**Keywords:** Cancer microenvironment, Diagnostics, Target identification, Imaging, Tumour biomarkers

## Abstract

Traditional pathology approaches have played an integral role in the delivery of diagnosis, semi-quantitative or qualitative assessment of protein expression, and classification of disease. Technological advances and the increased focus on precision medicine have recently paved the way for the development of digital pathology-based approaches for quantitative pathologic assessments, namely whole slide imaging and artificial intelligence (AI)–based solutions, allowing us to explore and extract information beyond human visual perception. Within the field of immuno-oncology, the application of such methodologies in drug development and translational research have created invaluable opportunities for deciphering complex pathophysiology and the discovery of novel biomarkers and drug targets. With an increasing number of treatment options available for any given disease, practitioners face the growing challenge of selecting the most appropriate treatment for each patient. The ever-increasing utilization of AI-based approaches substantially expands our understanding of the tumor microenvironment, with digital approaches to patient stratification and selection for diagnostic assays supporting the identification of the optimal treatment regimen based on patient profiles. This review provides an overview of the opportunities and limitations around implementing AI-based methods in biomarker discovery and patient selection and discusses how advances in digital pathology and AI should be considered in the current landscape of translational medicine, touching on challenges this technology may face if adopted in clinical settings. The traditional role of pathologists in delivering accurate diagnoses or assessing biomarkers for companion diagnostics may be enhanced in precision, reproducibility, and scale by AI-powered analysis tools.

## Introduction

**Fig. 1:**
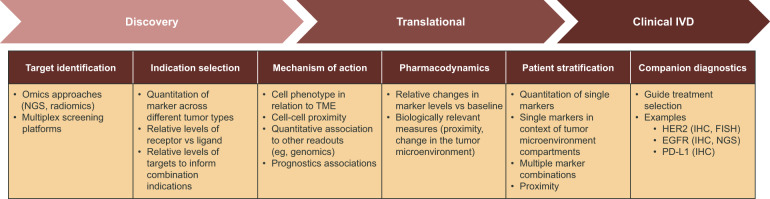
Digital pathology: from drug discovery to clinical diagnostics. Dx diagnostic, EGFR epidermal growth factor receptor, FISH fluorescence in situ hybridization, HER2 human epidermal receptor 2, IHC immunohistochemistry, IVD in vitro diagnostic, MOA mechanism of action, NGS next-generation sequencing, PD pharmacodynamics, PD-L1 programmed death-ligand 1, TME tumor microenvironment.

Pathology has historically played a crucial role in the drug development process, including preclinical research to facilitate target identification, define drug mechanism of action and pharmacodynamics, and enable toxicology assessments^1,2^. More recently, pathology has formed a bridge between drug discovery, translational, and clinical research programs that are striving to decipher disease pathophysiology in the context of the mechanism of action, patient selection, or patient stratification (Fig. 1)^3,4^. Such insights form the basis of novel hypotheses that can further be explored in drug discovery programs or applied to inform clinical trial design, thereby improving the probability of technical and regulatory success.

Pathology-based assessments have been used to classify disease and determine efficacy in drug development across a variety of disease areas^5–7^. For example, during phase 2 trials for drug development in non-alcoholic steatohepatitis, the US Food and Drug Administration (FDA) considers evidence of efficacy on a histological endpoint to support initiation of phase 3 trials^7^. Additionally, pathological complete response (pCR) has been studied as a surrogate endpoint in patients with cancer for the prediction of long-term clinical benefit and favorable prognosis with the administration of neoadjuvant therapy^8–13^. More recently, pCR was associated with improved long-term efficacy in patients with human epidermal growth factor receptor 2 (HER2)-positive breast cancer treated with chemotherapy plus either intravenous or subcutaneous trastuzumab^14^. In the immuno-oncology (I-O) arena, immune-related pathologic response criteria have been applied retrospectively to surgical specimens from patients treated with immunotherapy in the neoadjuvant or advanced disease setting to predict survival in several tumor types^15,16^.

Immunohistochemistry (IHC) has been used to characterize biomarkers, such as programmed cell death-ligand 1 (PD-L1), and their association with clinical benefit. Traditional pathology techniques present several advantages, such as low cost, widespread availability, and application on formalin-fixed, paraffin-embedded (FFPE) tissue samples^17^, but challenges pertaining to differences in laboratory methods and subjective interpretation, particularly with the evaluation of immune cell staining, may lead to inter-observer variability^18^. This can produce inconsistency in diagnoses, which may impact treatment decisions^19–23^. While the use of IHC assays has led to better identification of patients who respond to I-O therapy^24–26^, there remains a need to more accurately quantify complex immune markers, including cell phenotypes in a spatial context, that require advanced quantitative tools to maximize the amount of information yielded from individual samples^27,28^.

Artificial intelligence (AI) applications in pathology improve quantitative accuracy and enable the geographical contextualization of data using spatial algorithms. Adding spatial metrics to IHC can improve the clinical value of biomarker identification approaches. For example, in a recent meta-analysis, the addition of spatial context to IHC, achieved using multiplex IHC and immunofluorescence (IF), was significantly better at predicting objective response to immune checkpoint inhibitors (ICIs) compared with gene expression profiling (GEP) or IHC alone^28^, indicating the need for more complex computational approaches to decipher the underlying biology and enhance clinical utility.

The development and integration of digital pathology and AI–based approaches provide substantive advantages over traditional methods, such as enabling spatial analysis while generating highly precise, unbiased, and consistent readouts that can be accessed remotely by pathologists^29^.

## Advancing from traditional pathology to digital pathology

**Fig. 2:**
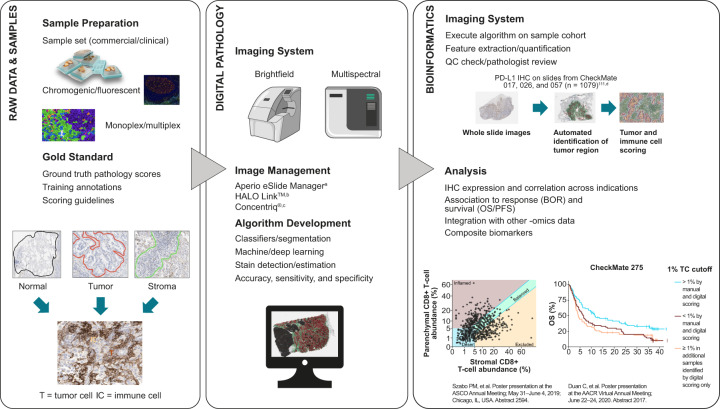
Digital prognostic pathology workflow. BOR best overall response, IHC immunohistochemistry, OS overall survival, PFS progression-free survival, QC quality control. ^a^Leica Biosystems; ^b^Indica Labs; ^c^Proscia; ^d^PD-L1 IHC 28-8 pharmDx. Dako/Agilent Technologies.

Efforts to overcome some of the challenges seen with traditional pathology methods have led to the development and adoption of complex, novel imaging systems and whole slide image (WSI) scanners that have enabled the transition of pathology into the digital era, also known as digital pathology. Within minutes, WSI scanners capture multiple images of entire tissue sections on the slide, which are digitally stitched together to generate a WSI that can be reviewed by a pathologist on a computer monitor (Fig. 2)^30,31^. Two scanners, Philips IntelliSite Pathology Solution (PIPS) (Philips, Amsterdam, Netherlands) and Leica Aperio AT2 DX System (Leica Biosystems, Buffalo Grove, Illinois, USA), are approved by the FDA for review and interpretation of digital surgical pathology slides prepared from biopsied tissue^32,33^.

There are many practical advantages to using these digital pathology image systems and solutions that would bring substantial benefits to translational and clinical research. These include the organization and storage of large amounts of data in a centralized location, integration of digital workflow software to help streamline processes and improve efficiency, convenient sharing of image data to enable cross-specialty worldwide remote communication, reduced testing turnaround time, and the generation of precise and highly reproducible tissue-derived readouts reducing inter-pathologist variability^29,34–37^. The increased speed and efficiency gained in image acquisition can enhance the downstream utilization options of traditional techniques such as hematoxylin and eosin (H&E), IHC, and in situ hybridization. These slides can be converted into a remotely available image within minutes and centrally reviewed by multiple pathologists from various sites^29^, with applications including education, research, consultation, and diagnostics^29^.

Recently, due to ongoing disruptions in relation to the COVID-19 pandemic, including remote working and restricted travel, digital pathology has been crucial in the continuation of clinical and academic research, as well as routine pathology services^38^. Without the need to transport glass slides and the ensuing logistical and safety concerns, central pathology review enables secure remote working^38^. Additionally, the utilization of digital images allows the generation of pixel-level pattern information, leading to expanded use of computational approaches that enable a quantitative analysis of WSIs^39,40^.

### Improvements gained from digital pathology: quantitative analysis of the WSI

The use of digital image analysis in pathology can identify and quantify specific cell types quickly and accurately and can quantitatively evaluate histological features, morphological patterns, and biologically relevant regions of interest (e.g., tumoral or peritumoral areas, relationships between different immune cell populations, areas of expression, presence of metastasis)^41,42^. Quantitative image analysis tools also enable the capturing of data from tissue slides that may not be accessible during manual assessment via routine microscopy. Additionally, performing similar tasks manually can require significant time investment and can be prone to human error, such as counting fatigue^43,44^.

### Expanding data capabilities: multiplex and multispectral imaging

**Fig. 3:**
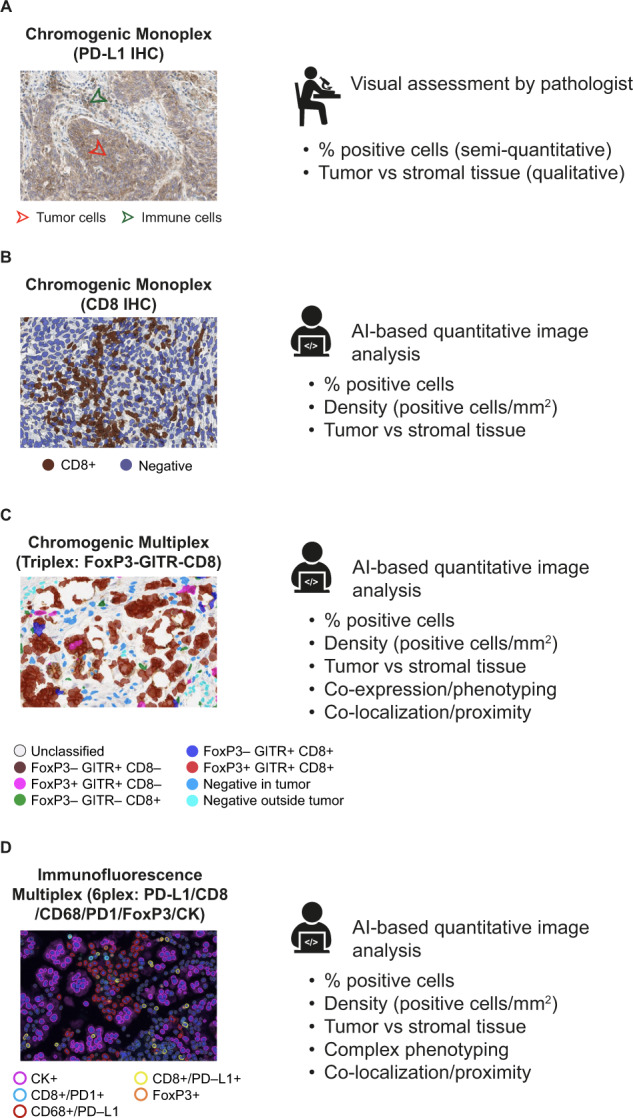
Applications of digital pathology in IHC. **a** Monoplex slide stained for PD-L1, as seen on a monitor. **b** Monoplex slide stained for CD8. **c** Multiplex stain annotated using AI-based analysis allowing multiple marker identification. **d** Multispectral immunofluorescence. Legend indicates examples of possible phenotypes detected from this assay; however, many more are possible. CD cluster of differentiation, PD-1 programmed death-1, PD-L1 programmed death-ligand 1.

Quantitative image analysis can also be used to generate high-content data through application to a technique known as multiplexing, which allows co-expression and co-localization analysis of multiple markers in situ with respect to the complex spatial context of tissue regions, including the stroma, tumor parenchyma, and invasive margin^45,46^. Current imaging metrics can utilize multispectral unmixing strategies to reveal co-expression patterns that define unique cell phenotypes and spatial relationships (Fig. 3)^47^.

Automated classification of epithelial and immune cells and simultaneous marker analysis at the single-cell level has been conducted using prostate cancer, pancreatic adenocarcinoma, and melanoma tissue samples^46,48,49^. Application of this technique allowed identification of distinct T-cell populations and their spatial distributions and underscored the potential of immune markers to identify patients who may benefit from immunotherapy^48,49^.

While a highly multiplexed imaging platform can be used to understand intra- and inter-cellular signaling pathways by examining how phenotypically distinct cell populations are spatially distributed relative to one another, it is a time-consuming process applicable to a predefined region of interest^50^. However, as technology quickly advances, allowing digital evaluation of entire tissue slides, we are no longer confined to a region of interest^37,51^. The wealth of new information provided by these techniques has created a need for more consistent and reproducible interpretation of large and complex datasets, along with defining the interaction patterns between cell types and spatial context found in pathological images that define biological underpinnings^37,52,53^.

### Advances in computational approaches: AI and machine learning

The need for data reproducibility and the increasing complexity of the analyses described above has led to the application of AI in pathology^37,52,53^. AI refers to a broad scientific discipline that involves using algorithms to train machines to extract information or features beyond human visual perception^37,41,54^. AI approaches are built to initially extract appropriate image representations and then to train a machine classifier for a particular segmentation, diagnostic, or prognostic task using a supervised or unsupervised approach^37,41,54^. The power of AI to analyze large amounts of data quickly can significantly speed up the discovery of novel histopathology features that may aid our understanding of or ability to predict how a patient’s disease will progress and how the patient will likely respond to a specific treatment^37,39,55^. In breast cancer, for example, unsupervised learning models have been used to generate histologic scores that can differentiate between low- and high-grade tumors and evaluate prognostically relevant morphological features from the epithelium and stroma of tissue samples to provide a score associated with the probability of overall survival^56,57^. The success of these AI-based approaches relies on the quality and quantity of the data used to train the algorithm, limiting the generalizability of these image analysis algorithms to larger or more complex datasets^58^.

#### Taking it further: deep learning networks

**Fig. 4:**
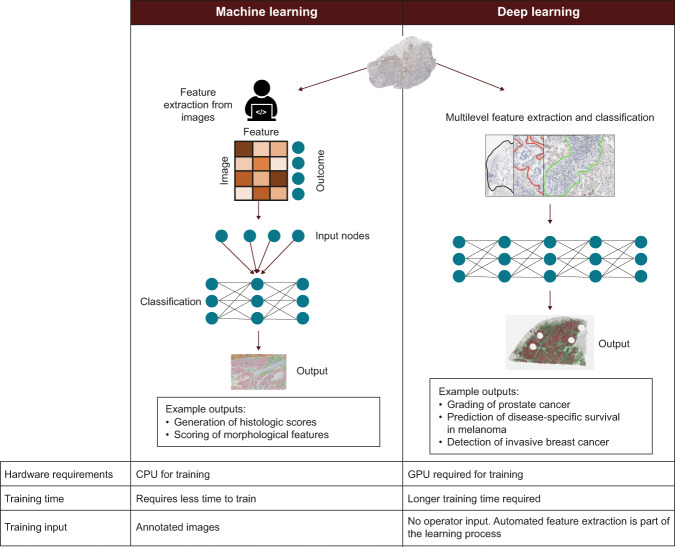
Comparisons between machine learning and deep learning. Deep learning is a subset of machine learning that uses multi-layer neural networks to analyze data, removing the need for operator input for feature extraction and image annotation^37,41,57,59–62,111,145^. CPU central processing units, GPU graphics processing units.

Deep learning takes machine learning a step further, using sophisticated, multilevel deep or convolutional neural networks (DNN or CNN) to create systems that perform feature classification from large datasets^37,40,41,54^. Figure 4 highlights key differences between machine learning and deep learning. The impact of applications of deep learning algorithms to IHC- and H&E-stained specimens have been well documented across many tumor types. These include grading prostate cancer^59^, identifying biomarkers for disease-specific survival in early-stage melanoma^60^, detection of invasive breast cancer regions on WSIs^61,62^, predicting response to chemoradiotherapy in locally advanced rectal cancer^63^, and identifying morphological features (nuclear shape, nuclear orientation, texture, tumor architecture, etc.) to predict recurrence in early-stage non-small cell lung cancer (NSCLC) from H&E slides^64^. Deep learning has also been used to construct entity-graph-based tissue representations, where cell morphology and topology are embedded within each node to effectively describe the phenotypical and structural properties of tissues and can be processed by graph neural networks (GNNs). GNNs therefore enhance the interpretability of pathological assessments gleaned from neural networks^65,66^.

It is important to compare AI-based interpretations with those of the pathologist to define the associated algorithm’s performance characteristics and utility. For example, when a CNN trained to classify melanoma samples was compared against manual scoring by histopathologists, the CNN was significantly superior in classifying images as malignant melanoma or benign nevi compared with manual assessment by histopathologists^67^. In the CAMELYON16 challenge, deep learning algorithms to detect breast cancer metastases in H&E-stained WSIs of lymph node sections performed similarly to the best performing pathologists under time constraints in detecting macrometastases and were better in detecting micrometastases^68^. However, it should be noted that the performance of any algorithm will depend on the task, due to the degree of accuracy required and the quality of the samples to be assessed^37^.

Another application of machine learning in the preclinical space is the assessment of tumor purity (TP). TP estimation, currently evaluated visually by pathologists, is used to ensure a signal is derived from cancer cells rather than other noncancerous cells that may be present in the TME based on tissue morphology when tissue is used to generate orthogonal data such as transcriptome or exome^69,70^. In a comparison of TP determined using AI (using deep learning algorithms generated on the PathAI platform) and manual estimates by pathologists, AI-assessed TP was found to be more accurate than visual assessment by pathologists^71^. Previous evidence has shown that immunosuppressive pathways are upregulated in patients with low TP, suggesting that low TP is associated with poor prognosis in some tumor types, including gastric cancer. Therefore, improved methods of evaluating TP may also aid in the identification of patients who may be suitable for immunotherapy^72^.

Given the amount of additional detail and insights that can be gained from combining WSI with machine learning algorithms, this technology can be readily applied to translational research. However, one major limitation of machine learning is the large amount of high-quality data required to develop these algorithms^58^. Data used for training need to be accurate and as complete as possible in order to maximize predictability and utility^39^. This can be challenging when histological data are obtained from various laboratories, leading to some variability due to factors such as differences in slide preparation (sectioning, fixation, staining, and mounting)^73^, scoring algorithms^18^, and inherent inter-observer variability^74^. These challenges become more apparent when more complex computational analytics methods are used for multiplexed imaging. Although AI could be used to overcome inter-reader variability across multiple institutions with the development of robust algorithms that take specific histological features of various tumors and subtypes into account^75^, further research is needed to fully understand the impact of these factors on the quality of AI data.

## Applications of digital pathology in translational medicine

### Enhancing our understanding of the TME

Tumor evolution and progression involve many complex cellulars and molecular interactions that are spatially and temporally regulated within the TME^52^. IHC can be used to gain insights into the composition of the TME by facilitating the identification of different cell types expressing a protein of interest and assessing the density and spatial distribution of specific biomarkers^50^. Digital pathology approaches, such as quantitative analysis of TILs, present an opportunity to gain greater insight into intra-tumor heterogeneity, spatial patterns of cell phenotypes, and the complex interactions between cancer and the immune system within the TME^52,53^. Image-based techniques can be used to determine immune cell responses to immunotherapy such as macrophage activation^76^ or lymphocyte infiltration by regulatory T cells (Tregs) into core tumor regions in solid tumors^77^, which may in turn have value as a predictive indicator for the effectiveness of ICIs. Favorable cancer prognosis has also been associated with factors in the TME, including high CD8^+^ TIL rates^78,79^. Recently, image analysis and AI methods have contributed to the development of novel approaches to concurrently assess multiple biomarkers in preclinical and exploratory studies, revealing complex interactions within the TME and providing the potential to improve cancer diagnosis and the selection of treatment regimens. Combining multiple techniques, such as multiplex IF, with image analysis has yielded important insights into specific immune cell populations, such as those in the TME of classical Hodgkin lymphoma, and their associations with PD-1/CTLA-4^+/−^ T cells^80^. These studies require multiple large cohorts to add the scale and robustness necessary to gain these important insights, to elucidate relationships that may not be apparent to the human eye, and to help overcome observer bias that may mask potential biomarker signals.

### Assessing treatment response: immune cell interactions in the TME

Digital pathology can also be used to gain insights into a receptor-ligand binding, as proximity may be indicative of receptor engagement and activation. For example, lymphocyte-activation gene 3 (LAG-3), expressed on exhausted T cells, principally interacts with major histocompatibility-II (MHC-II) molecules, expressed on the surface of antigen-presenting and tumor cells^81,82^. Spatial analysis in bladder and gastric cancer tumor cells has demonstrated that the density and proximity of LAG-3^+^ were significantly greater when associated with MHC II^+^ vs. MHC II^−^ tumor cells, suggesting that LAG-3–expressing TILs may be preferentially located in proximity to MHC II^+^ tumor cells, allowing for LAG-3 activation and the inhibition of antitumor immunity^83^. The insights provided by digital pathology into the number and location of immune cells relative to tumor cells may provide information on immune response^37,84^, which could guide future treatment strategies. AI has also been used to quantify immune cells within the TME to define T-cell abundance and associated geographic localization in the tumor stroma, parenchyma, parenchyma-stromal interface, and invasive margin, which are then associated with transcriptomic factors to define underlying biological associations^85^.

### Identifying genomic features

Additionally, AI-based approaches may find applications in translational medicine and clinical practice by predicting gene mutations from routine histopathology slides. With genomic tests being associated with high costs and high rates of failure due to stringent sample requirements^86,87^, AI may be particularly useful for evaluating genomic instability and the mutational landscape, with the possibility to assess pathologic and genomic features in conjunction with one another. A CNN trained with WSIs of H&E-stained hepatocellular carcinoma (HCC) tissue was used to predict the ten most common prognostic and mutated genes in HCC, with four of these (*CTNNB1*, *FMN2*, *TP53*, and *ZFX4*) correctly identified by the model^88^. Similar results were obtained when a DNN was trained to predict the most commonly mutated genes in lung adenocarcinoma, with 6 (*STK11*, *EGFR*, *FAT1*, *SETBP1*, *KRAS*, and *TP53*) being predicted from WSIs^89^. Deep learning has also been used to predict microsatellite instability (MSI) status from tumor tissue^90^. A CNN trained to classify MSI versus microsatellite stability was able to robustly distinguish features predictive of MSI in gastric and colorectal cancer samples^90^.

However, there are limitations to using AI for molecular classification. For example, current imaging techniques can only identify genetic variants when they directly impact tissue morphology, as described previously^91^. At the same time, AI algorithms cannot be applied in cases where actual variant allele frequencies of selected mutations can impact the classification and prognosis of individual diseases, such as hematologic myeloid neoplasms^92^.

## Translating digital pathology into clinical practice

### Potential for patient stratification

As a further application in translational medicine, digital pathology approaches have been used to predict response and identify patients most likely to respond to treatment. For example, studies have used spatial analysis to determine the response of patients with NSCLC to nivolumab therapy. These included training machine learning models to extract morphological details, such as the spatial arrangement of tumor nuclei and variance in shape and chromatin structure^93^, as well as the area and density of TILs and the proximity of TILs to each other and to tumor cells^94^. The features extracted from these models were able to distinguish patients who responded to nivolumab therapy^93,94^. In another example, digital image analysis was used to quantify CD8 and PD-L1 positive cell densities from patients treated with durvalumab across multiple tumor types^95^. Patients defined as positive for the CD8xPD-L1 composite signature had longer median survival compared with signature-negative patients, demonstrating the potential predictive value of digitally defined composite biomarkers.

AI and machine learning can also assist in classification and staging across various tumor types. A new approach to tumor subtyping has been developed based on a DNN (MesoNet) to predict OS of patients with mesothelioma from hematoxylin, eosin, and saffron stained WSIs, without any pathologist-provided annotations^96^. Results demonstrated that the model was more accurate in predicting patient survival than using current pathology practices and was able to identify regions contributing to patient outcomes^96^, suggesting that deep learning models can identify new features predictive of patient survival and potentially lead to new biomarker discoveries.

### Application of digital pathology and AI algorithms in diagnostics

Biomarker research has been an area of particular interest in the I-O space due to its potential predictive value in some solid tumors^25,26,97–100^. ICIs, such as anti–PD-(L)1 and anti–cytotoxic T lymphocyte antigen-4, have been studied in multiple clinical trials, leading to improved prognosis for patients across various solid tumors^101^. Evidence has shown that PD-L1 expression may be indicative of response to ICI therapy in some tumor types^25,26,97–100^, while other studies have shown that patients demonstrated durable responses to ICIs regardless of PD-L1 expression^3,102–108^. Given the widespread clinical use of ICIs, predictive assays are needed to help stratify patients to determine who may benefit from such treatments.

While the use of these assays can help determine whether a patient will benefit from ICI therapy, biomarker identification, such as PD-L1 status, using tumor biopsies is challenging. Even when used by experienced pathologists, visual interpretation of PD-L1 using IHC is subjective and prone to error, which may contribute to inaccurate patient stratification. Digital scoring of PD-L1 expression can assist pathologists in overcoming these barriers by providing standardized metrics for biomarker assessment at single-cell resolution across whole tissue sections^36^.

Multiple studies have evaluated PD-L1 assessment using digital scoring and AI algorithms and have shown that digital-based techniques can perform better than or equal to manual pathological evaluation across various tumor types. A high correlation between AI and manual assessment of PD-L1 expression on tumor and immune cells has been observed in multiple CheckMate trials with samples from NSCLC, urothelial carcinoma, melanoma, and gastric cancer^109–111^. Furthermore, similar associations between PD-L1 expression and response to nivolumab have been reported between manual and digital scoring^109,110^. Using the combined positive score to assess PD-L1 expression on tumor and immune cells, digital image analyses and pathologists’ interpretations on stained slides (using the 22C3 pharmDx assay [Dako, Denmark]) demonstrated 33 (84.6%) of 39 cases had concordant results, and statistical analyses indicated that PD-L1 expression interpreted by pathologists or digital image analysis did not differ significantly for predicting responses to pembrolizumab^112^. Prospective clinical trials in colorectal cancer^113^ and NSCLC^114^ are also using digital image analyses to identify potential immune cell biomarkers within the TME.

The role of AI and machine learning in biomarker identification has been evaluated in studies outside of immunotherapy. For example, a DNN model (ConvNets) trained to automatically recognize cancer cell types were compared with conventional machine learning techniques. ConvNets achieved significantly higher accuracy than conventional algorithms, suggesting a role for computer-aided diagnosis to facilitate clinical decision-making^115^. Beyond oncology, AI and machine learning have been studied in the context of a morphological assessment of nonalcoholic steatohepatitis/nonalcoholic fatty liver disease and liver allograft fibrosis^116,117^. In these cases, AI-based methods were able to correctly reflect markers of steatotic severity^116^ and assess liver allograft fibrosis progression over time^117^.

Various platforms have been developed for the purpose of quantitative image analysis. Several have received FDA approval, including those used to detect HER2^118^. The goal of a HER2-directed image analysis platform is to detect and quantify HER2 membranous IHC staining of invasive breast cancer cells and to provide an accurate, precise, and reproducible quantitative HER2 result that can then be used to guide treatment decisions^119^. Digital image analysis has also been used to classify biological subtypes beyond HER2, including ER- and progesterone receptor (PR)–positive subtypes. Ahern et al demonstrated considerable overlap between unsupervised and supervised computational pathology platforms using image analysis to measure ER and PR expression in breast tumors between positive and negative groups, as classified by a pathologist^120^. While the supervised platform had a marginally higher performance than the unsupervised platform, both platforms provided meaningful results and may have important roles in future molecular epidemiology studies^120^.

### Addressing consistency issues for application in clinical practice

There are several published resources for pathologists as well as for physicians, including guidelines, position papers, and directives relating to digital pathology^30,31,37,121–128^. These include detailed information on the handling of digital images in nonclinical^121^ and clinical^122^ settings, technical aspects and performance standards for WSI devices^122–124^, validation and quality assurance of digital pathology systems for nonclinical^125^ and clinical use^30,122,126^, AI concepts and best practices^37,127^, tutorials on using deep learning frameworks for image analysis^128^, and reimbursement considerations^129^. For example, the College of American Pathologists provides comprehensive guidelines to laboratories on validating their own WSI systems for clinical use, including emulation of the real-world environment, sample set size, establishing concordance using intra-observer variability, and documentation, among others^30^.

The performance of AI applications in digital pathology is largely dependent on the size and quality of the dataset used to train an algorithm^41^. Digital images used for training purposes should be obtained from multiple staining batches, scanners, and institutions to ensure generalizability. Such datasets should be curated by pathologists, ensuring that representative images have been obtained at an appropriate magnification and that all regions of interest are comprehensively annotated depending on the diagnostic application^41^. Crucially, the validation of AI algorithms developed for clinical purposes increases the concordance between manual and digital pathology interpretations. The role of pathologists in the validation step is equally important in order to ensure that datasets represent the sample type of interest (e.g., H&E-stained FFPE section), encompass the entirety of a glass slide, and are big enough to reveal potential interpretational discrepancies, as well as to evaluate the accuracy and performance of the algorithm^30,119^.

## Adoption of digital pathology and AI: challenges and future considerations

Despite the advantages of incorporating digital pathology into the clinical setting, challenges remain (Table 1). Value determination and reimbursement structures for digital pathology are lacking. This leaves value interpretation, investment, and cost savings considerations up to individual laboratories, which is difficult and a substantial hinderance to widespread adoption. Image analysis platforms have been shown to provide prognostic value, such as risk classification in patients with colon cancer^130^. However, these are offered as single-site, standalone tests, thereby limiting their applicability to the wider pathology community. Studies that have evaluated the adoption of complete digital pathology workflows have shown increases in efficiency and operational utility^131^.

**Table 1:** Advantages and limitations of digital pathology.

Feature	Advantages	Limitations
Data use and requirements	• Digital images and associated data can be made available to the wider community through electronic medical records^37,41,146^	• Success of AI-based approaches relies on the quality and quantity of the data used to train the algorithm^58^ • Data from DNN can be difficult to extract and interpret^41^, and algorithms can be slow to configure and run^39^
Clinical utility	• Ability to utilize automated algorithms to assist in identification and diagnosis, thereby reducing user error^37,41,146^ • Instantaneous viewing of high-resolution, true-color capture of sustained histology slides^37,41^	• Limited access to large, well-annotated datasets, which may limit clinical utility^39^ • Limited availability of AI-based devices with premarket regulatory approval^41^
Efficiency	• Ability to view multiple images at once across different magnifications^146,147^ • Efficient storage and management of digital slides and associated clinical information^146,147^	• Algorithm development is a time-consuming endeavor^148^
Cost	• Provides opportunities for better management of pathologist workflow^147^	• Financial cost of equipment, advanced software, and instrumentation^58^ • Reimbursement for the cost of AI-based methods is largely unknown^41,58^

Technical concerns related to reproducibility, interpretability, the accuracy of competing devices, financial costs of processing hardware, and regulatory approvals that must accompany studies of clinical utility all represent barriers to adoption^132^. Some level of error with digital pathology is anticipated to be present at this point, and approaches that combine algorithm performance with manual validation, with margins of error similar to or stricter than those used for manual pathology, are likely to be the standard moving forward. This approach has already been tested in routine diagnostics, whereby pathologists interacted directly with an AI platform to conduct IHC-based intrinsic subtyping of breast cancers. The AI platform, both alone and working in consort with pathologists, was significantly more accurate in determining subtypes^133^. Additionally, translation and adoption into clinical practice will depend on algorithms being validated across many patient cohorts utilizing data not included in the training set. This will require large amounts of data to be acquired from multiple laboratories in order to assure the broad applicability required in a clinical setting^39,134^.

While there have been instances of AI being used in the clinical trial setting, most have been observational studies^135^. Techniques that take into account variations in real-world practice and can influence decision-making need to be evaluated in interventional studies to ascertain true clinical value^134^. Although a protocol for the development of a reporting guideline and risk of bias tool has been published^136^, no official guidelines are available yet on the numbers of annotations, images, and laboratories needed to capture the variation seen in the real-world. Additional statistical studies will be required for application to properly determine the optimal processes and workflows to ensure full implementation of this technology in clinical practice^39^. Algorithms would also be subject to periodic quality assurance (eg, when a new staining protocol is introduced), similar to how assays are revalidated when there is a change in workflow or procedure^137^. Various quality control (QC) techniques can be used to overcome preanalytical issues such as variations in slide preparation, origin, and scanner type. One approach is to train individual models of the same architecture to recognize specific variables^73^. Other approaches, such as combining image metrics in a QC application^138^, or transformation of image patches with synthetically generated artifacts^139^, can be used to train an algorithm to recognize different types of histological artifacts. Other unforeseen hurdles may exist once these systems are in place, including unfamiliarity with a new system and associated need for training, technical support, security, monitoring, and software integration^140,141^. In the US, software solutions should be developed under the FDA’s Quality System Regulation and Good Machine Learning Practices. However, artificial neural networks have been described as “black boxes”, whereby data can be difficult to interpret, which may lead to regulatory concerns, as image features are extracted in ways that are difficult for a human to understand^127,142^. Despite the challenges, the efficiency gains, such as faster results and higher throughput, are key motivators for pathologists to adopt digital pathology.

The benefits of AI can be seen across all stages of the drug development process and in the clinical setting^143^. One of the first applications of AI in the clinical setting is likely to be assessing multiple IHC I-O markers within a single tissue section. Application of image analysis to multiplexed IHC–stained samples offers accelerated scan times while increasing accuracy and productivity by automatically measuring parameters that may be hard to reliably achieve by eye^47^.

In the evolving field of digital pathology, a strategy towards the implementation of digital pathology may involve several phases culminating in the adoption of digitized images and AI technology in the clinic. A first step involves demonstrating the reliability of digital pathology with a biomarker that has shown clinical utility with manual pathology, such as approved complementary diagnostics. For example, using PD-L1 expression, which has demonstrated clinical utility across a range of tumor types^26,100,144^, would allow digital pathology readouts to be compared directly with manual pathology data and clinical outcomes. In this phase, pathologists would maintain a role in QC, but with improved efficiency. Data from the evaluation of such biomarkers with digital pathology could then be used in applications to the FDA for companion diagnostic status. Subsequent steps would introduce digital pathology as a diagnostic with novel biomarkers, with the aim of demonstrating the clinical utility of the biomarker with digital quantification. This phase would require the development of AI-based software for use in prospective clinical trials to evaluate the selected biomarker for patient stratification or selection. The next phase, and the long-term goal of digital pathology, would be to establish deep learning AI models trained using large quantities of data^39^ that can predict patient response and stratify patients using only WSIs.

## Conclusions

The current advances in digital pathology offer practical advantages over manual pathology, including enhanced accuracy and precision, the ability for digital images to be uploaded and reviewed remotely by multiple pathologists, and the acquisition and processing of large and complex datasets. Within immuno-oncology, a deeper understanding of the complexity and underlying mechanisms of the TME can be achieved with the help of AI and machine learning, where datasets can be consistently analyzed and validated for application across many large cohorts, which may have implications for drug development and clinical trial design. AI and machine learning can then be utilized within the clinic to describe clinical and pathologic features across multiple patient samples. These advances will not only facilitate the entry of more precise I-O therapies, but also ultimately improve diagnostic, prognostic, and predictive clinical decision-making in cancer treatment.
